# Genome-Wide Identification and Characterization of SET Domain Family Genes in *Brassica napus* L.

**DOI:** 10.3390/ijms23041936

**Published:** 2022-02-09

**Authors:** Sarfraz Sehrish, Wahid Sumbal, Meili Xie, Chuanji Zhao, Rong Zuo, Feng Gao, Shengyi Liu

**Affiliations:** Key Laboratory of Biology and Genetics Improvement of Oil Crops, Oil Crops Research Institute of Chinese Academy of Agricultural Sciences, Ministry of Agriculture and Rural Affairs, Wuhan 430062, China; sehrishsarfraz04@gmail.com (S.S.); sumbalwahid@gmail.com (W.S.); xiemeili0101@163.com (M.X.); zhaochuanji@caas.cn (C.Z.); hu086zr@163.com (R.Z.); liusy@oilcrops.cn (S.L.)

**Keywords:** SET domain, histone lysine methyltransferases, gene structure, phylogenetic analysis, *Brassica napus*

## Abstract

SET domain group encoding proteins function as histone lysine methyltransferases. These proteins are involved in various biological processes, including plant development and adaption to the environment by modifying the chromatin structures. So far, the SET domain genes (*SDGs*) have not been systematically investigated in *Brassica napus* (*B. napus*). In the current study, through genome-wide analysis, a total of 122 *SDGs* were identified in the *B. napus* genome. These *BnSDGs* were subdivided into seven (I–VII) classes based on phylogeny analysis, domain configurations, and motif distribution. Segmental duplication was involved in the evolution of this family, and the duplicated genes were under strong purifying selection. The promoter sequence of *BnSDGs* consisted of various growth, hormones, and stress-related cis-acting elements along with transcription factor binding sites (TFBSs) for 20 TF families in 59 of the 122 *BnSDGs*. The gene ontology (GO) analysis revealed that BnSDGs were closely associated with histone and non-histone methylation and metal binding capacity localized mostly in the nucleus. The in silico expression analysis at four developmental stages in leaf, stem root, floral organ, silique, and seed tissues showed a broad range of tissue and stage-specific expression pattern. The expression analysis under four abiotic stresses (dehydration, cold, ABA, and salinity) also provided evidence for the importance of *BnSDGs* in stress environments. Based on expression analysis, we performed reverse transcription-quantitative PCR for 15 target *BnSDGs* in eight tissues (young leaf, mature leaf, root, stem, carpel, stamen, sepal, and petals). Our results were in accordance with the in silico expression data, suggesting the importance of these genes in plant development. In conclusion, this study lays a foundation for future functional studies on *SDGs* in *B. napus*.

## 1. Introduction

The nucleosome, which is made up of two DNA strands wrapped around histone proteins (octamer), is the most fundamental unit of eukaryotic chromatin material. The histone octamer consists of two copies of each H2A, H2B, H3, and H4 histone protein [1]. Various alterations to the N-terminus of these histone proteins, such as methylation, acetylation, phosphorylation, sumoylation, glycosylation, ubiquitination, and ADP-ribosylation, influence the gene expression epigenetically [2,3]. Besides the other modifications, methylation at the specific lysine residue of histone protein is one of the important epigenetic modifications that affect the transcriptional regulation process. A SET domain is found in most of the proteins involved in histone methylation and constitutes a SET domain methyltransferase family. All the histone lysine methyltransferases (HMKTases) contain a conserved SET domain except the HMKTase that catalyzes the methylation of H3K79 [4]. HMKTases, with the help of SET domains, facilitate the transfer of methyl group from cofactor S-adenosylmethionine (AdoMet) to histone 3 (H3) lysine residues at positions 4, 9, 27, and 36, and histone 4 (H4) at position 20 [5]. One, two, or three methyl groups can be found on each lysine. In plants and animals, dimethylation or/and trimethylation of H3K4 and H3K36 can lead to gene activation, whereas transcriptional inactivation is caused by dimethylation of H3K9 and trimethylation of H3K27 [5]. Non-histone proteins, such as the rubisco, are also methylated by these SET domain-containing methyltransferases [6].

The SET domain group (SDG) protein family is named after the conserved regions of three proteins, initially identified in Drosophila. These three proteins are SUPPRESSOR OF VARIEGATION (Suv), enhancer of zeste E(Z), and TRITHORAX (TRX). The underlined letters constitute the name “SET”. The SET domain consists of approximately 130 to 150 amino acids. There are two parts of the SET domain, designated as SET-N and SET-C. Along with SET-N and SET-C, the overall structure includes an insert region (SET-I) with substantial structural diversity in flanking regions [7]. The SET-N has nine cysteine residues and is known as pre-SET; Zn atoms bind to them and stabilize the structure, whereas the SET-C consists of three cysteine residues, known as post-SET, which also participate in the zinc-binding site. Both flanking N and C terminal sequences of the SET domain facilitate methyltransferase activity [8]. In *Arabidopsis thaliana* (*A. thaliana*), the *SDG* gene family is subdivided into seven classes based on the domain architecture, function, and the presence of other domains along with the SET domain. These classes are (I) enhancer of zeste E(Z) homologs that methylate H3K27, (II) ASH1 (trithorax group protein) homologs that methylate H3K36, (III) trithorax homologs and related proteins, methylate H3K4, (IV) proteins PHD domain along with SET, methylate H3K4, (V) suppressor of variegation (Su(var)) homologs and relatives that methylate H3K9, (VI) interrupted SET domain-containing proteins with SET and myeloid-Nervy-DEAF-1 (SYMD), methylate H3K36, and (VII) rubisco methyltransferase (RBCMT) and other SET-related proteins (SETD) involved in non-histone methylation. Four classes, E(z), Ash, Trx, and Suv, are considered to be the principal classes [9,10]. It has been observed that numerous processes have been implicated in determining the regulation and mode of action of these SET domain-containing proteins [10].

*SDG* gene family has been identified in many plant species including *A. thaliana* [11], *Brassica rapa* [12], *Populus trichocarpa* [13], *Vitis vinifera* [14], *Zea mays* [15], *Oryza sativa* [16], *Solanum lycopersicum* [17], *Citrus sinensis* [18], *Litchi chinensis* [19], *Gossypium raimondii* [20], *Setaria italic* [21], *Triticum aestivum* [22], and *Dendrobium catenatum* [23] with 49, 67, 59, 33, 43, 43, 52, 47, 48, 52, 53, 166, and 44 members, respectively.

Until now, several *SDG*s are functionally characterized in plants. During plant development, these *SDGs* were implicated in a variety of biological activities [24,25,26,27,28,29,30,31], such as *MEA* and *SWN* are required for the development, dormancy, and germination of Arabidopsis seeds [29,32,33]. *ASHH2* participates in shoot branching, *ashh2* mutants developed additional shoot branches than wild Arabidopsis plants due to reduced histone H3K4 and K36 methylation [34]. *ATX1* maintains the root architecture system from regulating the cell production to cell elongation in root apical meristem [35], whereas the *CLF* is also involved in vegetative growth such as root and leaf development [36] in Arabidopsis plants. *SDGs* have a significant impact on reproductive development as well. *ATXR3* controls the pollen development and female gametophyte development [37], while the overexpression of *ASHR3* or *ATXR5–6* leads to male sterility through methylation regulation of H3K4 and K36 [26,38]. *SDGs* influence flowering via altering the histone methylation levels of flowering time genes. For instance, *ATX1* and *ATXR3* are the repressor of *FLC* and deposit the H3K4me3 marks at the *FLC* locus to control flowering time in Arabidopsis [39,40,41]. Additionally, *CLF* also maintains the repressive mark at H3K27me by PRC-complex regulation and controls flowering through *FLC* inactivation [42]. The *ashh2* mutant induced early flowering via *FLC* repression in *A. thaliana* and *B. napus* [30,43]. The *SDGs* take part in the activation of gene expression as well; for example, *SUVH1*, *SUVH3*, *SUVH7*, *SUVH8* prevent the DNA hyper-methylation at approximately 1000 genomic places through the regulation of *ROS1* gene expression [44]. The *SETD* genes cause trimethylation of rubisco and regulate the carbon fixation process during the Calvin cycle [45]. The *SDGs* are also involved in response to external cues, including both abiotic and biotic stresses [21,22,37,46,47,48,49]. *ATX1* facilitates the H3K4me3 modification and activates the ABA biosynthesis gene named *NCED3* to improve Arabidopsis drought tolerance [47]. *ASHH2* activates the defense-related genes against *P. syringae* in tomato plants [50]. These findings indicate that the *SDGs* alter the histone methylation signals and affect the growth and adaptation of plants.

*Brassica napus L*. (*B. napus*) is one of the essential oilseed crops around the world and is formed by the recent hybridization of *Brassica rapa* and *Brassica oleracea* [51]. To date, the *SDG* gene family has not been reported in *B. napus*. In the current project, we identified 122 *BnSDGs*. In addition to physical mapping to chromosomes, evolutionary analysis, gene, and protein structure analysis, promoter cis elements along with transcription factor binding sites prediction and gene ontology (GO), we also analyzed their tissue and developmental stage-specific expression pattern behaviors from publically available RNA sequencing data and verified potential 15 candidate genes in eight tissues through quantitative reverse transcriptase PCR. The detailed characterization of these genes broadened our knowledge of *SDGs* in *B. napus*.

## 2. Results

### 2.1. Identification of SDGs in B. napus

In the current study, we identified 122 *BnSDGs* in the *B. napus* genome, and each gene was named after its homolog in *A. thaliana* [9,12]. The *BnSDG* homologs were given the same name and differentiated by using A and B, according to the localization of genes on the A or C subgenome, respectively. If more than one *SDG* homologs belonged to one *At*SDG, they were differentiated by numbers 1, 2, 3... such as *BnASHH3.1A*, *BnASHH3.1B*, *BnASHH3.2A*, *BnASHH3.2B*. We also identified 65 and 37 homologs of *BnSDGs* in *B. rapa* and *B. oleracea*, respectively. These genes were also named in the same way, and more than one homolog for one *A. thaliana* gene was differentiated by using alphabets, a, b, c... such as for *B. oleracea*, *BoASHH3a*, *BoASHH3b*, and for *B. rapa*, *BrASHH3a*, and *BrASHH3b*. SET domain was present in all the identified SDGs.

The homologs for *BnaA05g17880D*, *BnaCnng44980D*, and *BnaA09g52100D*, *BnaCnng01720D* were not annotated as *SDGs* in *A*. *thaliana* (*At1g33400*, *At1g43245*), as reported in the previous report [12]. The detailed information of 122 *BnSDGs* is described in Table 1. A total of 64 genes belong to the A subgenome, whereas 58 genes belong to the C subgenome (Table 1). The gene length varied from 1149 (*BnASHR2.1A*) to 10,285 bp (*BnATXR3.1A*) with the presence of 1–26 exons per gene. The CDS length varied from 774 to 6897 bp, and the protein length varied from 257 to 2298 amino acids. The isoelectric points were ranged from 4.49 to 9.55 (Table 1). Moreover, the GRAVY (grand average of hydrophobicity) index was ranged from −0.876 to −0.027. The subcellular localization results revealed that 94 proteins were localized to nuclear regions, 15 proteins were localized in the cytoplasmic region of the cell, 7 proteins were found in the extracellular region, and the remaining fraction of proteins were specifically located in the mitochondrion, plasma membrane, endoplasmic reticulum (Table 1).

### 2.2. Phylogenetic Analysis of BnSDGs

**Figure 1:**
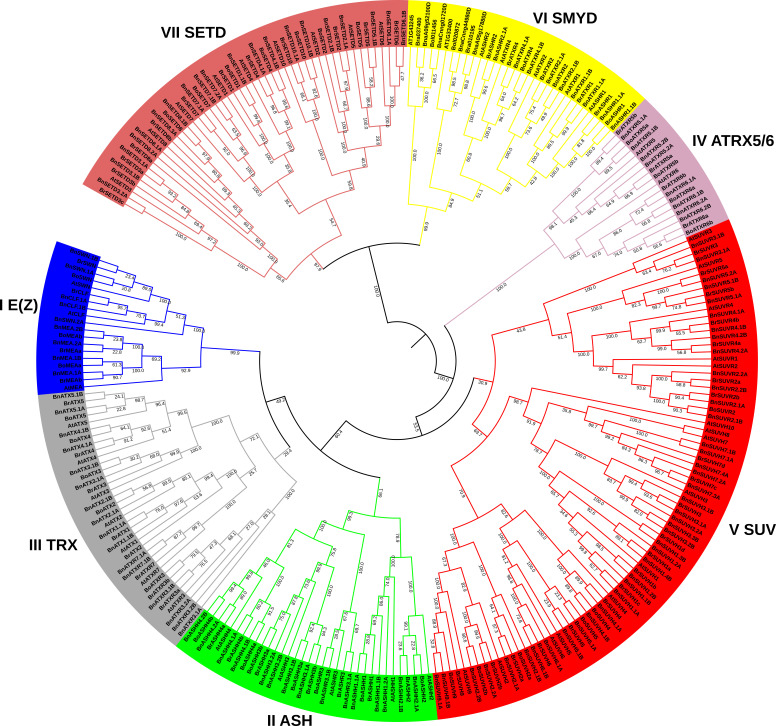
A neighbor-joining (NJ) phylogenetic tree of SDG proteins based on SET domain sequence between *Brassica napus* (*B. napus*), *Brassica rapa* (*B. rapa*), *Brassica oleracea* (*B. oleracea*), and *Arabidopsis thaliana* (*A. thaliana*). The SDGs were grouped into seven classes (I–VII) with 1000 bootstrap replication. Each class is represented by a unique color code. All the nodes represent bootstrap values.

To further characterize and find the evolutionary relationship of BnSDGs with *A. thaliana* and *B. napus* ancestral species (*B. rapa* and *B. oleracea*), we constructed a phylogenetic tree, using NJ (neighbor-joining) method with SET domain sequence of 122 BnSDGs, 49 AtSDGs, 65 BrSDGs, and 37 BoSDGs (Figure 1). The BnSDGs were assigned to I–VII classes based on their sequence homology with SDGs with *A. thaliana*, *B. rapa*, and *B. oleracea* with well-supported bootstraps indication. We found that 9, 15, 16, 8, 41, 11, and 22 BnSDGs belonged to Classes I–VII, respectively. The four principal groups (E(z)), Ash, Trx, and Suv contained a total of 40% (81/122) BnSDGs (Figure 1). Suv class comprised of largest group SET domain-containing SDGs among all the other groups. The homologs of newly identified AtSDGs At1g33400 and At1g43245 were evolutionarily related to Class VI members. All the identified classes were subcategorized into one (Class IV) to seven (Class V) orthology groups (Table S2).

### 2.3. Assignment of BnSDGs to Chromosomes and Synteny Analysis

**Figure 2:**
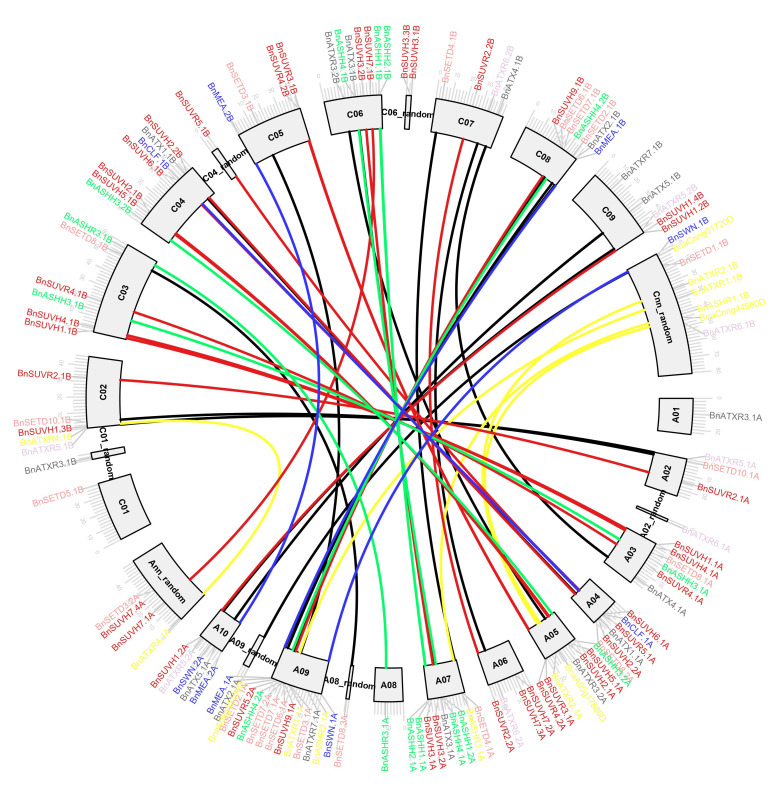
Location of *SDGs* on *B. napus* chromosomes. The gene names are mentioned outward of each chromosome. The scale bar represents Mb. The Class I E(z), II Ash, III Trx, IV ATXR5/6, V Suv, VI SMYD, VII SETD *BnSDGs* are represented by blue, green, gray, purple, red, yellow, and rust color, respectively. The connected lines between the chromosomes represent the duplicated gene pairs.

We investigated the physical location of *SDGs* by analyzing the distribution of the genes on the *B. napus* chromosomes (Figure 2). The results showed that 122 *BnSDGs* are distributed on all 19 chromosomes and the random fragment chromosomes. A total of 64 *BnSDGs* were located in the A subgenome whereas, 58 genes were located on the C subgenome. There was no special distribution arrangement on the chromosomes for all the I–VII classes of *BnSDGs*. Moreover, chromosome A09 showed a maximum number of genes (14), and chromosome Cnn displayed a maximum of eight genes in the C subgenome. Each of A01, A08, A09_random, A02_random, A08_random, C01, C01_random, C04_random chromosomes contained a single *SDG* gene. As Class V is the largest class, its members were distributed on all the chromosomes except chromosome A01, C01, and Cnn. Most of the genes were located at the terminal regions of the chromosomes; only a few were distributed near the centric regions (Figure 2).

**Figure 3:**
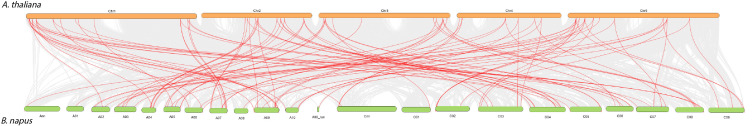
Collinearity analysis of *SDGs* between *B. napus* and *A. thaliana*. Background gray lines show the collinear blocks within *B. napus* and *A. thaliana* genomes, whereas the red lines represent the syntenic *SDG* pairs.

The collinearity analysis releveled the strong orthologous relationship of *SDG* genes between *B. napus* and *A. thaliana* (Figure 3, Table S3). Our results showed that 20, 21, 16, 12, and 24 *BnSDGs* were collinear with chromosomes 1, 2, 3, 4, and 5 of *A. thaliana*, respectively. All the Class IV *BnSDGs* showed syntenic relation with chromosome 5 only, whereas Class III and Class VII *BnSDGs* had shown collinearity with all the five chromosomes of *A. thaliana*. These results suggest that *BnSDGs* have sustained the syntenic blocks with the ancestor plant (*A. thaliana*) and the expansion of the *SDGs* could be the result of duplication events in *B. napus*.

Duplication events are one of the key evolutionary processes that can lead to structural and functional differentiation [52]. We performed a blast between the 122 *BnSDGs* CDS sequences and obtained a total of 49 duplicated pairs based on their sequence similarities (>80%) (Table S4). Our results demonstrated that segmental duplications have played an important role in the expansion of *SDGs* in the *B. napus* genome. Moreover, we detected nine dispersed duplication events, including in two gene pairs (*BnASHR1.1A*, *BnASHR1.1B*, and *BnaA05g17880D*, *BnaCnng44980D*), three tandem, one interspersed, and one proximal gene duplication type (Table S4). Ka (non-synonymous substitutions)/ Ks (synonymous substitutions) is a significant parameter that is used to determine the selection pressures during evolution [53]. To understand the evolutionary pressure on *BnSDG* duplicates, the Ka, Ks, and Ka/Ks ratios for all the 49 duplicated gene pairs were determined. Our results revealed that all of the duplicated *BnSDGs* duplicated genes had a Ka/Ks ratio of <1 except for two pairs *BnATX5.1A*, *BnATX5.1B*, *BnASHH3.1A*, and *BnASHH3.1B*, suggesting that the *BnSDGs* were under strong purifying selection during the evolution (Table S4).

### 2.4. Structural (Domain and Motif Conservation) and Functional Analysis (Gene Ontology (GO)) of BnSDGs Proteins

To characterize and analyze the structural variations in all BnSDGs, we performed a conserved domain analysis (Table S5). Our results revealed that BnSDG proteins contained other important domains along with the SET domain. These additional domains included CXC, AWS, pre-SET, post-SET, WIYLD, PWWP, PHD, FYRC, FYRN, Zf, TPR-like, and YDG/SRA (Table S5). Based on the presence of specific domain/s architecture, all the BnSDGs were distributed into seven classes (I–VII). Class I had a CXC domain to the N-terminus of the SET domain in all the proteins (Table S5). Class II showed the AWS domain to the N-terminus of the SET domain and a post-SET domain toward the C-terminus. BnASHR3.1A, BnASHR3.1B, BnASHH1.1A, BnASHH2.1A, and BnASHH2.1B had extra ZF domain to the N-terminus of the AWS domain (Table S5). Class III showed maximum domains in the proteins. ATX related protein BnATXR3.1A, BnATXR3.1B, BnATXR3.2A, BnATXR3.2B, BnATXR7.1A, BnATXR7.1B contained only SET and LRR and DUF/GYF_2 domains. BnATX1.1A, BnATX1.1B, BnATX2.1B, and BnATX2.1A contained PWWP, FYRN, FRNC, PHD, zf-HC5HC2H_2, SET and post-SET domains. BnATX3.1A, BnATX3.1B, and BnATX4.1A had PHD, PWWP, FYRN, FRNC PHD_2, zf-HC5HC2H_2, SET, post-SET in the direction of N- to C-terminus of the proteins. BnATX5.1B has a similar domain combination. Only the PHD domain was absent (Table S5). In Class IV, all the members contained the PHD domain to the N-terminus of the SET domain. The characteristic domains for SUV homologs were YDG/SRA, pre-SET, SET, and post-SET, whereas, in SUV-related proteins, instead of YDG/SRA, the WILD domain was present along with other above-mentioned domains. Only BnSUVR5.1A, BnSUVR5.2A, and BnSUVR5.1B have lost the WILD domain and acquired zf-TRM13_CCCH and zf-C2H2. The post-SET domain was lost in several members of this class (Table S5). The Class VI proteins contained the interrupted SET domain and additionally contained zf-MYND and TPR-like domain (Table S5). In Class VII, in a total of 20 genes, 15 SDGs (homologs of SETD1, SETD3, SETD4, SETD7, SETD8, SETD10) encoded for rubisco large sub-unit N-methyltransferases, whereas 5 BnSDGs (SETD2, SETD5, and SETD6) encoded for rubisco small sub-unit N-methyltransferases (Table S5).

We also performed the conserved motif analysis for each of the seven classes (I–VII) (Table S6). Based on the similarities in the sequences, the BnSDGs contained common motifs in each class and many unidentified motifs (Table S6). Overall, the conserved domains and motif within the BnSDGs proteins classes (I–VII) also supported their phylogenetic relationships.

**Figure 4:**
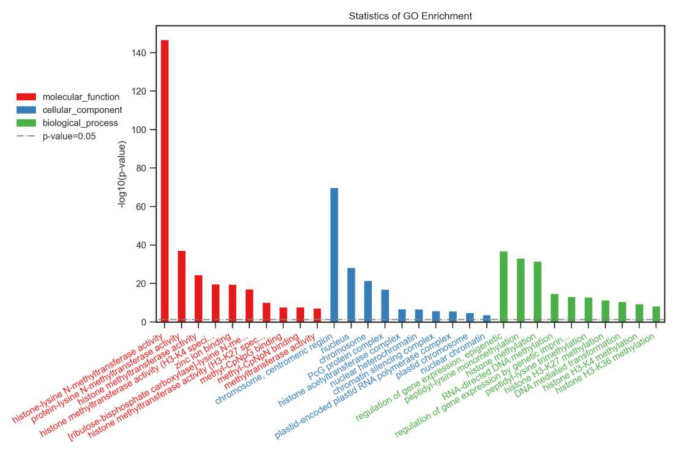
Gene ontology enrichment analysis of BnSDG proteins based on biological processes, molecular function, and cellular component.

We performed gene ontology (GO) annotation and enrichment analysis to predict the functions of the SDGs in *B. napus*. The identified several GO terms were classified into three categories: molecular function (MF), biological process (BP), and cellular component (CC) (Figure 4). The molecular function (MF) included mainly the histone lysine methyltransferase activity. The remaining GO terms broadly predicted the non-histone (RUBISCO) methylation, Zn binding capacity, and DNA/protein binding functions (Figure 4). The diverse biological processes (BP) were identified in this analysis, that included vegetative growth (shoot formation, leaf morphogenesis, seed dormancy, embryo sac development), reproductive growth (floral organogenesis, stamen development, carpel development, ovule development), stress responses (DNA repair, response to chitin, light stimulus), programmed cell death, cell differentiation, and organelle organization of chloroplasts and ribosomes. The GO cellular component (CC) terms demonstrated that BnSDGs were mainly part of nuclear regions. The remaining proteins were related to the chloroplast, endoplasmic reticulum, PcG protein complex, cytoplasm, plasma membrane, and plasmodesmata. The CC GO terms were consistent with subcellular localization information of BnSDG proteins (Table 1).

### 2.5. Gene Structure and Promoter Analysis of BnSDGs

**Figure 5:**
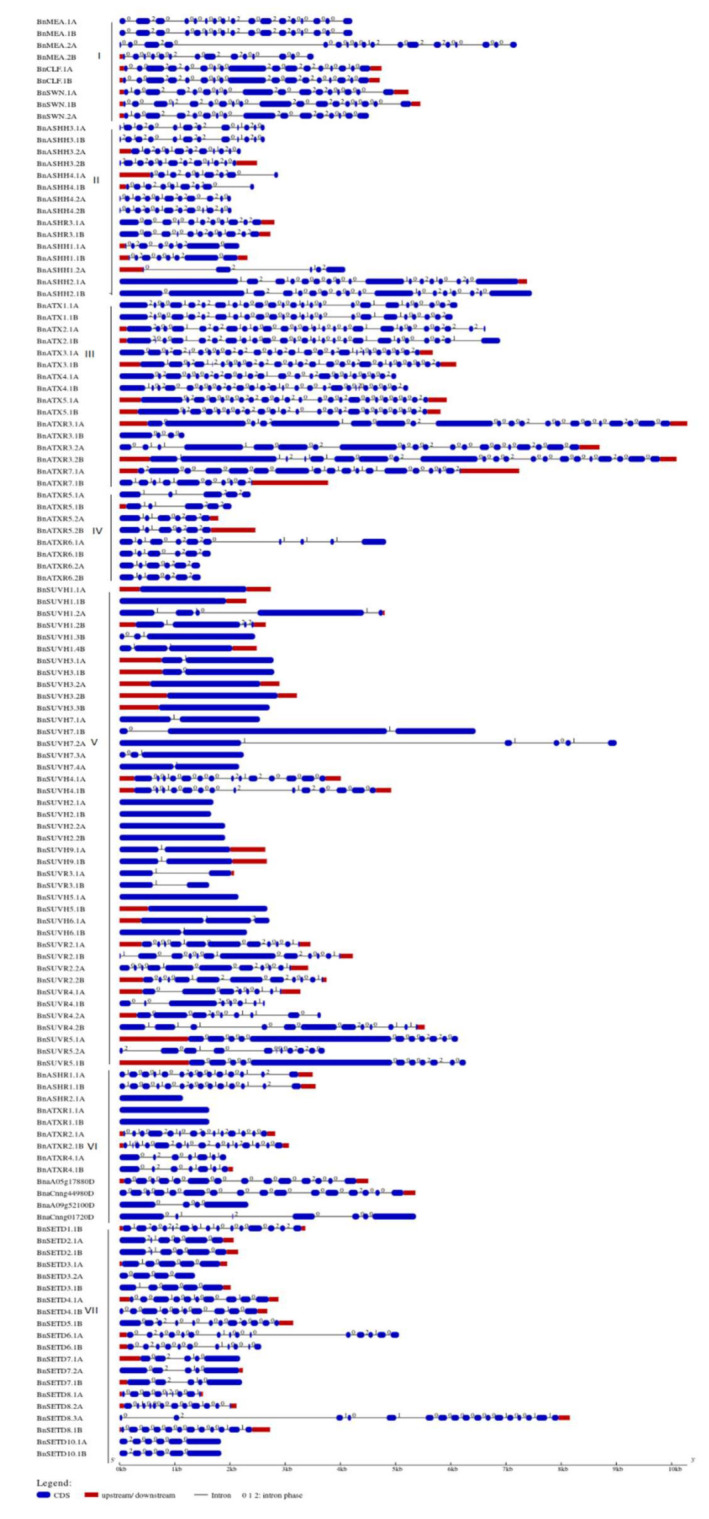
Exon-intron architecture of the *BnSDGs*. The genes are ordered according to classes (I–VII). Red boxes and blue boxes represent the untranslated region (UTR) and exons, respectively. The introns are shown by the black lines. The introns phases (0, 1, 2) are mentioned above each intron. A value of 0 means intron is between the two codons, 1 means intron is located after the first base of a codon, 2 means the intron location after the second base of a codon. The scale bar represents the gene size.

The exon-intron structures of *BnSDGs* were investigated to determine the structural evolution in the *BnSDG* gene family. The findings suggested that there was considerable variation in the number of exons (1–26) and introns (0–25) in individual *BnSDGs*. The numbers of introns and exons were relatively variable within the same class as well (Figure 5, Table 1) except Class I, which consisted of 16–18 exons and 15–17 introns. A total of 11 out of 41 *BnSDGs* in Class V and 3 *BnSDGs* in Class VI were intronless, whereas Class III had the maximum number of exons and introns (Figure 5, Table 1). Among all the introns in the *BnSDGs*, the distribution of phases was 56.17%, 22.3%, and 21.3% for phases 0, 2, and 1, respectively (Figure 5).

**Figure 6:**
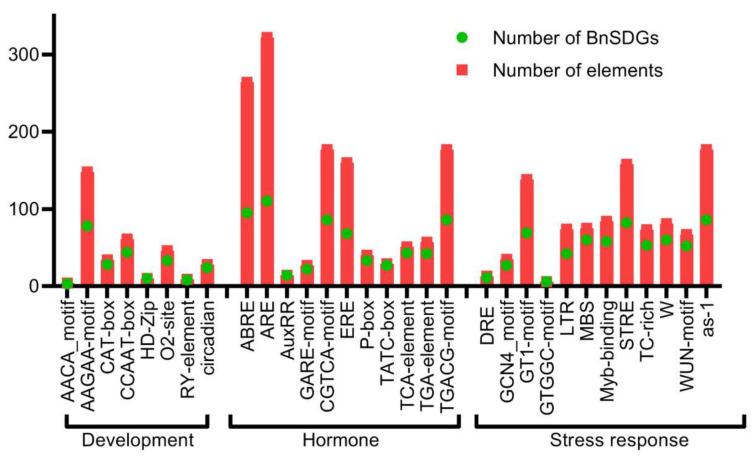
Cis-acting regulatory elements in the promoters of *BnSDGs*. The cis-acting elements were mainly categorized into developmental, hormonal, and stress-responsive elements. The bar graph indicates the total number of each cis-acting element found in *BnSDG* promoters (red box), as well as the number of *BnSDG* promoters that include a specific cis-regulatory element (green circle). Table S10 contains detailed information.

We identified the potential cis-regulatory elements in the 2 kb upstream coding region of *BnSDGs* by using the PlantCARE database. The promoters of two *BnSDGs* (*BnATXR3.1B* and *BnATXR3.2A*) were excluded from this analysis because the sequences of these promoters were undetermined. We obtained several kinds of cis-regulatory elements, including basic transcription regulators (TATA box, CAAT box) and condition-specific elements related to development, hormone, and stress response (Table S8). Moreover, some elements were present in multiple numbers in one promoter sequence. We broadly categorized them into three groups, i.e., growth/development, hormone-responsive, and stress-responsive elements (Figure 6, Table S9). Interestingly, there were 19 types of light-responsive elements, and the most commonly present light elements were box, G-box, and TCT elements. B-Box was found in 94 (78%) of all the gene promoters. The other growth-related elements included the AACA motif, AAGAA-motif, CAT-box, CCAAT-box, HD-Zip, O2-site, RY-element, and circadian (Tables S9 and S10) that are important during endosperm development, palisade mesophyll cells differentiation, seed, meristem, compound metabolism, and circadian clock regulation. These elements were randomly distributed in *BnSDGs* (Figure 6, Tables S9 and S10). The hormonal responsive elements included ABRE (abscisic acid-responsive element), AuxRR (auxin-responsive element), ERE (ethylene response element), CGTCA-motif (methyl jasmonic acid-responsive), GARE-motif, P-box and TATC-box (gibberellin-responsive element), TCA-element (salicylic acid-responsive element), TGA-element (auxin-responsive element). The abscisic acid-responsive elements were found in maximum copies and detected in 95 (78%) gene promoters, followed by methyl jasmonic acid-responsive elements in 86 (72%) *BnSDG* promoters (Figure 6, Tables S9 and S10). The stress-responsive elements included ARE (anaerobic induction), DRE (dehydration stress-responsive elements), MBS (MYB binding site involved in drought inducibility), LTR (low-temperature response), GC-motif (anoxic specific), WUN-motif (wound-responsive element), TC-rich repeats (defense and stress-responsive elements) and as−1 (pathogenic related) (Figure 6, Tables S9 and S10). Among all the stress elements, anoxic-specific elements were present in 110 (92%) *BnSDG* promoters. The most abundant potential cis elements were anoxic specific followed by light specific B-box and abscisic acid-responsive elements (Figure 6, Tables S9 and S10). These results suggest the importance of *BnSDGs* in development and stress conditions.

We analyzed the transcription factor binding sites (TFBSs) in the promoter (2 kb upstream of coding region) of 120 *BnSDGs* and identified 59 *BnSDGs* with TFBSs corresponding to 20 transcription factor families (Table S11). These transcription factors included GATA, MYB, C2H2, MIKC_MADS, Dof, Trihelix, BBR-BPC, AP2, B3, bZIP, ERF, bHLH, G2-like, CPP, MYB related, SRS, NAC, E2F/DP, ARF. Among all, 28 and 31 promoter sequences had single and multiple TFBSs, respectively. Furthermore, 12 promoter sequences showed two while the remaining promoters showed up to five TFBSs (Table S11).

### 2.6. In Silico Differential Expression Analysis

**Figure 7:**
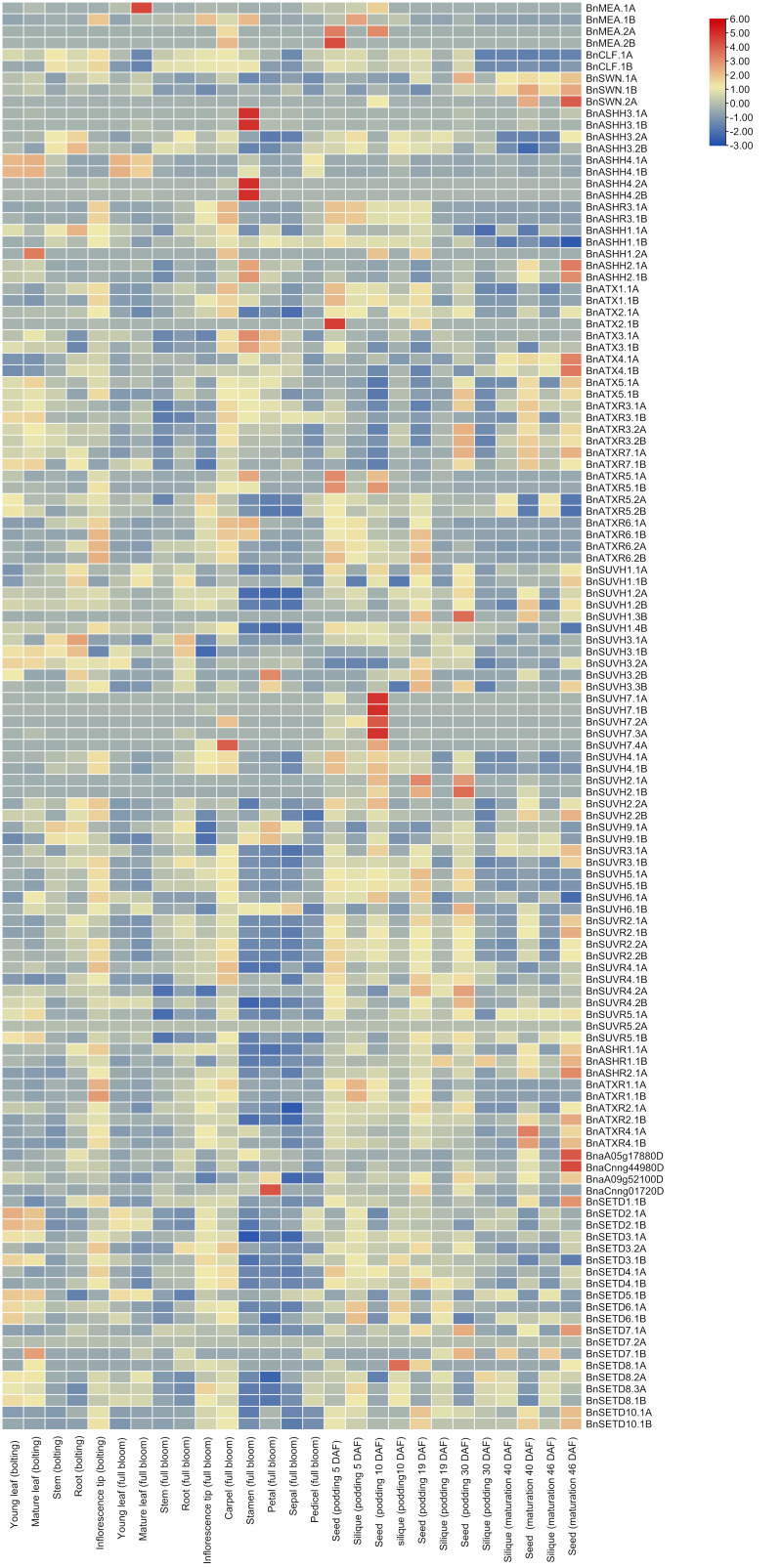
In silico expression analysis of *BnSDGs* in various tissues at bolting, full bloom, podding, and maturation stages of *B. napus*. DAF indicates days after flowering. The heatmap was generated based on row scale and by taking log2 fold of fragments per kilobase million (FPKM).

The recently launched, comprehensive Brassica Expression DataBase (BrassicaEDB) was used to extract the expression data at four developmental stages (bolting stage, full bloom stage, podding, and maturation) in different tissues to analyze the importance of *BnSDGs* during the development in *B. napus*. The tissues included were (a) young leaf, mature leaf, inflorescence tip, stem and root at the bolting stage, (b) young leaf, mature leaves, stem, root, inflorescence tip, petal, sepal, stamen, carpel, and pedicel at the full bloom stage, (c) and (d) seed and silique at podding and maturation stage, the time for seed and siliques collection was 5, 10, 19, 30, 40 and 46 days after flowering (Figure 7, Table S12). Our results demonstrated that *BnASHH4.1A* of Class II displayed the highest FPKM value (127) in the mature leaf at the bolting stage. The other Class II *BnSDGs* showed comparatively high FPKM values and stage-specific gene expression (*ASHH3. 1A*, *ASHH4. 2A*, *ASHH3. 1B*, *ASHH4. 2*) in stamen at the full bloom stage. Class V SUVH homologs 1, 2, 3, and 9 showed high FPKM and differential expression at all developmental stages, whereas the SUVH7 homologs were not active (threshold FPKM 0.5) except *SUVH7. 4A* in carpel and seed at the podding stage. Generally, in the case of Class I, *CLF* and *SWN* homologs were active at all developmental stages, except *BnSWN.2A*. Among all the *MEA* homologs, only *BnMEA. IB* was active in bolting, bloom, and podding stages. Class III and VI ATX and related homologs showed seed-specific expression at the maturation stage. Class III *BnSDGs* were expressed differentially at all the stages except *BnATX2.1B*. Class IV *BnSDGs* were predominantly expressed in podding stage (Figure 7, Table S12). Class VI proteins with interrupted SET domain were predominantly expressed in carpel and inflorescence tip at bolting and bloom stage and seed in maturation stage. In Class VII, *BnSETD5.1B* displayed the highest FPKM in the young, mature leaf and siliques at podding and maturation stage, whereas *BnSET7.2A, BnSET7.1B*, and *BnSET8.1A* were inactive in all the data. Due to the diverse range of expression patterns, the FPKM values were transformed to log2 fold for better visualization of differential expression across all the *BnSDGs* through heatmap (Figure 7). Our expression analysis revealed that *BnSDGs* might be involved in several stages and tissue-specific developmental processes.

### 2.7. In Silico Abiotic Stress Expression Analysis

During the stress conditions, the growth and development of *B. napus* have been greatly influenced. To determine the importance of *BnSDGs* in abiotic stresses, we used the transcriptome data of dehydration, cold, ABA, and NaCl treatments. Our results showed that the expression pattern of a total of 36 *BnSDGs* was changed by ± two-fold under the above-mentioned stresses (Figure S1, Table S13). Fewer genes showed greater fold change in response to ABA and NaCl treatments, whereas more genes showed significant change with response to cold treatment at 24-h. Among all these 36 genes, most of the genes were downregulated. A total of 22 *BnSDGs* were significantly responsive at 24 h cold stress (−6.3 to –2.8), and among these genes, 8 genes were upregulated. Only two *BnSDGs* showed a response (upregulated; 2.8 and 2.9) at only 4 h ABA treatment. A total of 5 *BnSDGs* were downregulated at 24 h NaCl treatment (−7.3 to –2.3). At 8 h dehydration, 7 genes (6 genes; downregulated), while at 1-hr dehydration, 11 genes (8 genes; downregulated) showed significant fold change expression. We did not find any *BnSDGs* response at 24 h ABA and 4 h NaCl treatment. Only two homologs *BnASHH4.IA* and *BnASHH4.1B* were responsive during dehydration, cold, and NaCl treatment (Figure S1, Table S13)

### 2.8. The Expression Validation by Reverse Transcription-Quantitative PCR

**Figure 8:**
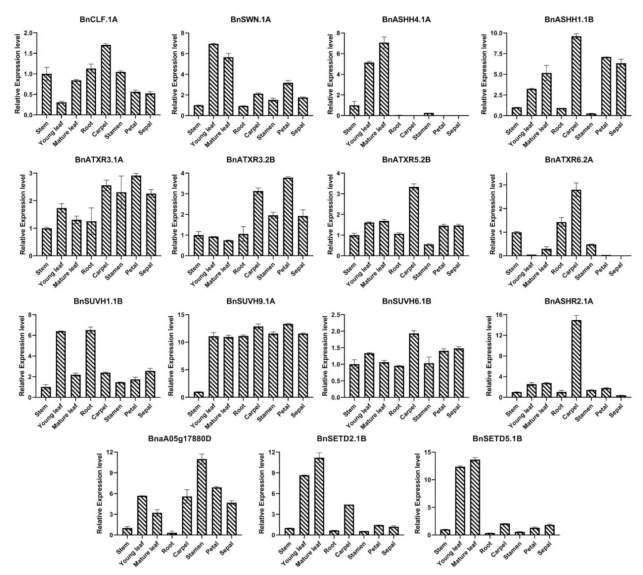
Expression profiling of 15 *BnSDGs* in eight tissues by quantitative reverse transcriptase PCR. The results were analyzed by using the 2^−ΔΔCT^ method. The error bars represent the standard deviation among the three biological replicates for each tissue.

To validate the in silico expression, we selected 15 *BnSDGs*, two from each class and three from Class V members*.1A*, *BnSWN.1A*, *BnASHH4.1A*, *BnASHH1.1B*, *BnATXR3.1A*, *BnATXR3.2B*, *BnATXR5.2B*, *BnATXR6.2A*, *BnSUVH1.1B*, *BnSUVH9.1A*, *BnSUVH6.1B*, *BnASHR2.1A*, *BnSETD2.1B*, and *BnSETD5.1B)*, based on the highest FPKM in each class using reverse transcription-quantitative PCR. The primers used in this analysis are mentioned in Table S1. The target genes expression was investigated in young leaf, mature leaf, stem, root, petal, sepal, carpel, and stamen at the full bloom stage. All the genes showed variable expression patterns in the tissues used (Figure 8). Seven genes (*BnCLF.1A*, *BnASHH1.1B*, *BnATRXR5.2B*, *BnATRXR6.2A*, *BnSUVH9.IA*, *BnSUVH6.1B*, *BnASHR2.1A*) were highly expressed in the carpel, whereas the *BnSWN.1A*, *BnSETD2.1B*, *BnSETD5.1B*, and *BnASHH4.1A* in young and mature leaf than in other tissues, and there was a variation of expression pattern within the members of the same class. For example, in Class II, the expression of *BnASHH4.1A* was predominant in leaf tissues with the least expression in flower tissues, but *BnASHH1.1B* was highly expressed in the carpel. We also isolated the cis-regulatory elements for these candidate genes (Figure S2) to find evidence for their variable expression pattern. The cis-regulatory elements included hormone-responsive elements, light-responsive elements, circadian control elements, meristem and seed-specific elements, ethylene, abscisic acid, and salicylic acid-responsive elements, and Meja responsive elements (Figure S2). We extracted the potential function and cellular components information of these predominantly expressed candidate genes by GO analysis (Table S7). The results revealed that the potential molecular function of the two *SETD* candidate genes is a non-histone methylation process, while the remaining genes displayed histone methyl transferase activity. The cellular component for *SETD. 1B* was chloroplast while nucleus and chromosome showed five and seven candidate genes, respectively (Table S7). These results showed that these candidate genes might have a functional role in the growth and developmental processes.

## 3. Discussions

The SET domain-containing proteins (SDGs) are the known histone lysine methyltransferases and participate in several developmental and physiological processes [11,31]. So far, there is no detailed genome-wide investigation of SDGs in *B. napus*; therefore, the current study will facilitate new insights into this gene family and predict the potential function in plant growth and stress conditions.

In the present study, we identified 122 *SDGs* in the *B. napus* by using the *Darmor*-*bzh* v4.1 genome sequence information. The brassica lineage has gone through whole-genome triplication after the separation from *A. thaliana*. Moreover, the *B. napus* is an allopolypolypliod that is a product of the hybridization of *B. rapa* and *B. oleracea* [51,54]. Therefore, the six times increase was expected in *BnSDGs*. However, the identified *BnSDGs* were lower in number. We also found that almost all of the *AtSDGs* have one to two homologs in both A and C subgenomes, suggesting that many *SDGs* are lost due to diploidization events in the genome. It is noteworthy to mention that *AtSUVH8*, *AtSUVH10*, *AtSUVR1*, and *AtSETD9* had no orthologs in the *B. napus*. Their orthologs were also not found in *B. rapa* and *B. olerecea* except for *AtSETD9*, which had one ortholog in the *B. rapa* (Table S2). These results showed that the loss of these genes occurred after the separation of brassica lineage. However, the number of *SDGs* is greater in *B. napus* as compared to previously identified *SDGs* in species such as Arabidopsis (49), rice (34), maize (43), foxtail millet (53) [10,15,21]. Until now, the maximum number of *SDGs* (166) are detected in hexapolyploid wheat crop [22], suggesting that duplication events within the polyploid crops played a crucial role in the expansion of *SDGs* during evolution. Our results showed that segmental gene duplication played a significant role in the evolution of *BnSDG* genes (Table S4). The Ka/Ks analysis showed that the duplicated gene pairs were under strong positive selection (Table S4). These duplication events led to genome expansion and functional diversity in the organisms [52].

According to phylogenetic analysis, the identified *BnSDGs* were placed into seven classes (I–VII), and the names were assigned according to the previous nomenclature used in *A. thaliana* and other species [9,12]. Thus, the *BnSDGs* were classified into seven classes along with *AtSDGs*, *BrSDGs*, and *BoSDGs* genes (Figure 1, Table S2), suggesting the close evolutionary relationship between four related plants species. These classes (I–VII) also possessed one to several orthology groups as per the previous studies [12,22]. Notably, the homologs *BnaA05g17880D* and *BnaCnng44980D*; *BnaA09g52100D* and *BnaCnng01720D* of unannotated *AtSDGs* (*At1g33400* and *At1g33400*) were placed in Class VI that contained interrupted SET domain. The arrangement of *BnSDGs* in the phylogenetic tree was further verified by the gene and protein/domain structure analysis. The domain analysis verified the arrangement of these *BnSDGs* in the phylogenetic tree. The characteristic domains of each class were conserved (Table S5) [12,22]. A total of 102 *BnSDGs* were histone methyltranferases whereas, the remaining 20 out of 122 were SET-related proteins that might be involved in the methylation of non-histone proteins such as rubisco. All the homologs of *SETD1*, *3*, *4*, *7*, *8*, and *SETD10* were rubisco large sub-unit N-methyltransferases encoding genes, whereas *SETD2*, *5*, and *SETD6* were rubisco small sub-unit N-methyltransferases encoding genes. Except for BnSETD3.2A and BnSETD7.1A, all BnSETD proteins had a complete SET domain, in contrast to *A. thaliana*, which possessed a truncated SET domain in these SDGs, showing that the evolution of this polyploid crop enabled the structural variations in these *BnSDGs* [10]. The structural analysis of *BnSDGs* has provided information about gene length variation, ranging from 1.149 to 10.285 kb. Their corresponding proteins vary in length from 257 to 2298 amino acids. These results are also validated by *SDGs* in other plants species [15,21,22] and maize has the longest gene length (44 kb) [15] known among all the *SDGS* detected. However, the high percentage occurrence of 0 intron phase (56.17%) in *BnSDGs* signifies the conservation of coding sequences, as described previously [12]. The introns were absent in all the homologs of *SUVH2*, *SUVH3*, *SUVH5*, and two homologs of *SUVH1*. These results are consistent with *A. thaliana*-respective *SDGs* homologs [55] except for one *SUVH1* homolog in *B. napus* that has four introns (Table 1).

Almost all the *BnSDGs* were localized in the nuclear region, and only a few were localized in cytosol endoplasmic reticulum, chloroplast, mitochondrion, and plasma membrane (Table 1). The previous studies also validated their presence in the nuclear region due to their involvement in epigenetic regulation [21]. These findings were also consistent with the gene ontology (GO) analysis (Figure 4). The GO analysis also predicted that the main function of *BnSDGs* was the addition of methyl group to histones, particularly and few non-histone proteins. Histone methylation plays a wide variety of roles in plant life, including vegetative growth, root and shoot development, reproductive organ development, and responses to external stresses [31]. However, the functional studies of *BnSDGs* are still lacking in *B. napus* except a few [30]. Therefore, their possible functions could be identified by analyzing the expressions profile of these *BnSDGs* in various tissue at several developmental stages. Our expression data analysis illustrated the spatiotemporal expression of *SDGs* at four developmental stages in various tissues (Figure 7, Table S12) of *B. napus*. Generally, the genes showed predominant expression in young and mature leaves followed by the reproductive organs such as carpel and stamen and a few genes with predominant expression in the maturation stage (Figure 7, Table S12). There was also the expression variation within the members of a class. The Class I and V (SUV) *SDGs* were expressed comparatively higher at many stages in several tissues but with the variable FPKM values, showing the differential expression pattern throughout the studied stages. In a previous report, the *clf* mutant plant showed curled-up leaves, abnormal root growth, and floral development in *A. thaliana* [31]. Similar expression variations were observed in leaf, root, and inflorescence of *BnCLF* homologs. *BnCLF.1A* showed relatively high expression in leaf and root as compared with *BnCLF.1B* at the bolting stage, which also shows the expressional divergence between the homolog genes. Moreover, the previous studies in *A. thaliana* showed that the over expression of *ATXR5* caused male sterility, and the effect of *ATXR6* overexpression was lethal for stamen development [38]; similarly, in our results, the homologs of *BnATXR5/6* expressions were lower in stamen tissues as compared to other tissues studied. *ASHH2* consisted of two homologs, *BnASHH2.1A* and *BnASHH2.1B*. Their comparative expressions were high in stamen tissue at the full bloom stage. The previous study also revealed that the mutant *ashh2* showed abnormal pollen growth in *A. thaliana* [25]. Both homologs of *ATX1* showed higher expression in the carpel as compared to other tissues studied at the full bloom stage. It validated that the loss of the *ATX* gene resulted in abnormal carpel growth [56]. Likewise, the expression of *ATXR3* homologs was higher in stamen, carpel, and seed tissues except for one homolog *ATXR3. 1B*, and these results were consistent with the previous report in *A. thaliana* [57]. The previous studies also showed that several *SDGs* were involved in expression regulation during biotic and abiotic stress [47,50]. We also analyzed the transcriptome data of several abiotic stresses, including dehydration, cold, ABA, and salinity (NaCl) treatments in *B. napus*, which revealed the differential expression of *BnSDGs* under these external cues (Figure S1, Table S13). Our expression analysis showed that 36 *BnSDGs* showed gene expression change by ± two-fold under the selected stresses (Figure S1, Table S13). This evidence suggests that *BnSDGs* can not only play a role in tissue and developmental stage-specific time but also during stress management. We selected 15 *BnSDGs* genes (two from each class and three from Class V) on the basis of predominant expression within a class. We observed their expression pattern in young leaf, mature leaf, stem, root, stamen, carpel, sepals, and petals through reverse transcription-quantitative PCR. Our results verified the variable expression pattern in various tissues (Figure 8).

The promoters of *BnSDGs* were also studied for the presence of growth, development, and stress-related cis elements (Figure 6 and Figure S2). We identified plant growth hormone (such as abscisic acid, ethylene, gibberellin, auxin, and MeJA responsive elements), several types of light-responsive elements (for example; G-box and circadian elements), seed-specific elements (for example; O2-site and Ry elements), and meristem-specific elements in the candidate genes (Figure S2, Tables S8 and S9). The previous studies also showed that these cis elements are of functional importance in plant growth and development. The light-dependent expression of *GRP7* was controlled by the circadian cis element in the promoter sequence [58]. Deletion of the G-box element reduces the promoter activity toward several stimuli’s including light and hormone responses [59]. The mutation in abscisic acid response elements not only lessens the ABA response but also can inhibit the leucine zipper proteins binding that might affect many biological responses [60]. The Ry and gibberellin elements are investigated grain quality in rice [61]. The occurrence of these cis-regulatory elements provided evidence of *BnSDGs*’s role in growth and development.

## 4. Materials and Methods

### 4.1. Identification of SET Domain-Containing Genes (SDGs) in Brassica napus (B. napus)

To identify the SET domain-containing genes (*SDGs*) in *Brassica napus (B. napus)*, we performed BLASTP (blast protein) and hidden Markov model (HMM) analysis. For BLASTP search in *B. napus* proteome available at BnaOmics database (https://www.bnaomics.xyz/blast, accessed on 9 October 2021), we used *A. thaliana* SET domain-containing protein sequences as a query and set the e-value 1e−5. The protein sequences of AtSDGs were retrieved from The Arabidopsis Information Resource-10 (http://www.arabidopsis.org/, accessed on 9 October 2021). For HMM analysis, the local HMMER 3.1 webserver (http://www.hmmer.org/, accessed on 9 October 2021) was used to search the putative SDGs with default parameters, and SET domain PF00856 was used as a query from the Pfam database (http://pfam.xfam.org/, accessed on 9 October 2021).

We further confirmed the presence of the SET domain in predicted SDGs by using the Pfam database (http://pfam.xfam.org/, accessed on 9 October 2021), SMART (http://smart.embl-heidelberg.de/, accessed on 9 October 2021), and conserved domain database (CDD)-Batch search tool (https://www.ncbi.nlm.nih.gov/Structure/bwrpsb/bwrpsb.cgi, accessed on 9 October 2021). The redundant SDGs were excluded manually.

The identified *BnSDGs* in *B. napus* were used to find their homologs in its parental species, i.e., *Brassica rapa*; *BrSDGs* and *Brassica oleracea*; *BoSDGs* through Brassicaceae Database (BRAD) (http://brassicadb.cn/, accessed on 9 October 2021) by using the above-mentioned methods and were further confirmed for the presence of SET domain by the same above-mentioned method.

All the BnSDGs sequences, i.e., gene, CDS, proteins, and promoters, were collected from genome data files of *B. napus* genome available at BnaOmics database (https://www.bnaomics.xyz/, accessed on 9 October 2021), and for *BrSDGs*, *BolSDGs*, and *AtSDGs*, all the sequences information was retrieved from respective genomes data files available at Brassicaceae Database (BRAD) (http://brassicadb.cn/, accessed on 9 October 2021).

### 4.2. Phylogenetic Analysis

The SET domain amino acid sequence was used to perform the phylogenetic analysis of SDGs in *B. napus*, *B. oleracea*, *B. rapa*, and *A. thaliana*. The multiple sequence alignment was carried out using CLUSTAL W with default parameters and some custom alignment in the MEGA version 7 program. MEGA7 was also used to generate the phylogenetic tree using the neighbor-joining (NJ) technique and pairwise deletion with 1000 bootstrap replicates. iTOL v6 (https://itol.embl.de/, accessed on 12 October 2021) was used for the final phylogenetic tree display.

### 4.3. Chromosomal Location, Synteny Analysis, and Ka/Ks Ratio

To physically map the *BnSDGs* on the *B. napus* genome, the location of genes and length of chromosomes were retrieved from the gff3 annotation file in the TBtools software, v1.098 [62]. Then the chromosomal location of *BnSDGs* was performed by advanced CIRCOS Tool in TBtools version 1.098 [62].

MCScanX was used to examine the collinearity correlations between the *B. napus* and *A. thaliana* genomes. The syntenic analysis of *SDGs* in *B. napus* was carried out against *A. thaliana* and visualized by using the dual synteny visualization tool in TBtools, v1.098 [62].

The ratios of synonymous substitution rate (ks) and non-synonymous substitution rate (ka) of homologous gene pairs were evaluated using TBtools, v1.098 [62] to assess if the *BnSDGs* encoding sequences are under selection pressure during the evolution. A Ka/Ks ratio of less than one indicated purifying selection, a Ka/Ks ratio of more than one indicated positive selection, and a Ka/Ks ratio of zero indicated neutral selection. T = Ks/2R, where R is 1.5 10^−8^ synonymous substitutions per site per year, was used to calculate divergence time 10^−6^ million years ago (MYA) [63].

### 4.4. The Biophysical, Structural and Functional Analysis of BnSDG Proteins

The biophysical properties of BnSDGs, such as molecular weight (MW), isoelectric points (IP), and GRAVY were determined by ExPASy-ProtParam tool (http://us.expasy.org/tools/protparam.html, accessed on 9 October 2021). The subcellular localization of BnSDG proteins was predicted CELLO v2.5 (http://cello.life.nctu.edu.tw/, accessed on 9 October 2021) [64].

The detailed domain structures, their start and end location in every protein were examined by using the Pfam database (http://pfam.xfam.org/, accessed on 9 October 2021), CDD-Batch search in NCBI (https://www.ncbi.nlm.nih.gov/Structure/bwrpsb/bwrpsb.cgi, accessed on 9 October 2021) and Interpro database (https://www.ebi.ac.uk/interpro/search/sequence/, accessed on 9 October 2021).

The conserved motifs of every class of BnSDG encoding proteins were scanned using the local MEME Suite v5.0.3 (https://meme-suite.org/tools/meme, accessed on 9 October 2021). For this objective, the following parameters were calibrated: maximum 10 motifs, with an optimal width of 6–50 amino acids. The remaining parameters were set to their default values. The identified motifs were annotated by using the Interpro database (https://www.ebi.ac.uk/interpro/search/sequence/, accessed on 9 October 2021).

The BnSDGs were functionally annotated using the Web server (https://cloud.oebiotech.cn/task/, accessed on 9 October 2021). Gene ontology (GO) terms were classified into biological process cellular component and molecular function.

The exon-intron structure and the intron phase (0,1,2) identification analysis were carried out by Gene Structure Display Server (GSDS 2.0) (http://gsds.cbi.pku.edu.cn, accessed on 9 October 2021).

### 4.5. The Identification of Cis-Regulatory Elements and Transcription Binding Sites in Promoter Regions

For the cis-elements analysis, 2 Kb upstream regions of the coding region of *BnSDGs* were examined by using the PlantCARE database (https://bioinformatics.psb.ugent.be/webtools/plantcare/html/, accessed on 12 October 2021). We also predicted the transcription factor binding sites (TFBSs) in the promoter region of *BnSDGs* by using the PlantRegMap/PlantTFDB v5.0 (http://plantregmap.gao-lab.org/binding_site_prediction.php, accessed on 12 October 2021).

### 4.6. In Silico Expression Analysis of BnSDGs

Brassica Expression DataBase (BrassicaEDB), v1.0 (https://brassica.biodb.org/, accessed on 12 October 2021) was used to analyze the expression data of *BnSDGs* at bolting (tissues used were; inflorescence tip, stem, root, young leaf and mature leaf), full bloom developmental stage (tissues used were; young and mature leaves, stem, root, inflorescence tip, stamen, petal, carpel, pistil, and pedicel), podding and maturation stage (tissues used were; seed and silique tissues at 5, 10,19 and 30 days after flowering). The FPKM values are converted to log2 fold, and heatmap was generated by using TBtools v1.098 [62]. For abiotic gene expression, data of dehydration, cold, ABA, and salinity [65] was used to calculate the log2 fold change, and later, a heatmap was generated.

### 4.7. Plant Material, RNA Extraction, and Reverse Transcription-Quantitative PCR

The *B. napus* v. ZS11 was grown in the field of Oil Crop Research Institute (OCRI) Wuhan, China. The samples of tissues were collected at the blooming stage. The tissues included young and mature leaves, stem, root, petal, sepal, carpel, and stamen. The samples were immediately put in liquid nitrogen and stored at −80 °C. Later, total RNA was isolated from the tissue samples using TRIZOL reagent (Invitrogen). Complementary DNA was synthesized using the PrimeScript RT Reagent Kit with genomic DNA Eraser (Takara) according to company instructions. The reverse transcription-quantitative PCR (RT-qPCR) reactions were carried out in three replicates using the SYBR green super mix (Bio-Rad). The reaction was set as 95 °C for 3 min, next 40 cycles of 95 °C for 10 s, ~58–60 °C for 30 s, and melt curve analysis at 65–95 °C, increment 0.5 °C for 0.5 s. β-Actin gene was used as an internal control, and Primer Premier v5.0 was used to synthesize the primers. The list of all the primers used in this study is included in Table S1. The results were analyzed using the 2^−ΔΔCT^ method as described previously [66]. The graphs were generated by using GraphPad Prism v8.0 [67].

## 5. Conclusions

Several publications have been documented in recent years, revealing that SET domain proteins are encoded by a vast multigene family in plants. In this study, we identified 122 *SDGs* in *B. napus* by using genome-wide analysis. Based on the evolutionary closeness and structural similarities, these *BnSDGs* were classified into I–VII classes. To understand their potential functional role, their evolutionary history, structure, cis-regulatory elements in the promoters, gene ontology (GO), and expression at various developmental stages were analyzed. Our results explained that gene loss and gene duplication both played a key role in the evolution of *SDGs* in *B. napus*. In silico expression analysis of *BnSDGs* revealed differential expression in different developmental stages, indicating that these genes play a role in plant development. Predominantly expressing 15 genes selected from each class were analyzed by RT-qPCR also revealed spatiotemporal expression. Cis-regulatory elements of these methyltransferases have growth, development, and stress-related elements. Overall, this study will help to better understand the complexity of *BnSDGs* and is beneficial for future experimental research on epigenetic regulation in *B. napus*.

## Figures and Tables

**Table 1:** A summary of detailed characteristics of *SDG* in *Brassica napus*.

	Transcript ID	Gene Name	Class	Chromosome: Location Start: Location End: Strand	Gene Size (bp)	CDS (bp)	Protein Length (aa)	PI	GRAVY	No. of Exons-Introns	Cellular Localization
1	BnaA09g51240D	*BnMEA.1A*		A09:33715032:33719248:−	4217	1932	643	6.3	−0.77	17–16	Nuclear
2	BnaC08g46050D	*BnMEA.1B*	I	C08:38367870:38372089:−	4220	1995	664	6.71	−0.757	17–16	Nuclear
3	BnaA10g01220D	*BnMEA.2A*		A10:640151:647345:+	7195	1917	638	8.65	−0.71	18–17	Nuclear
4	BnaC05g01310D	*BnMEA.2B*		C05:686198:689713:+	3516	1332	443	8.61	−0.671	15–14	Nuclear
5	BnaA04g13630D	*BnCLF.1A*		A04:11524589:11529333:+	4745	2724	907	9.05	−0.876	17–16	Nuclear
6	BnaC04g35880D	*BnCLF.1B*		C04:37428287:37432998:+	4712	2733	910	9.07	−0.851	17–16	Nuclear
7	BnaA09g00500D	*BnSWN.1A*		A09:243397:248630:+	5234	2559	852	5.76	−0.757	17–16	Nuclear
8	BnaCnng01170D	*BnSWN.1B*		Cnn_random:1341220:1346669:−	5450	2571	856	5.78	−0.797	18–17	Nuclear
9	BnaA10g10150D	*BnSWN.2A*		A10:8730712:8735228:+	4517	2526	841	5.61	−0.707	16–15	Nuclear
10	BnaA03g20680D	*BnASHH3.1A*	II	A03:9804836:9807462:+	2627	1110	369	5.35	−0.507	12–11	Nuclear
11	BnaC03g24710D	*BnASHH3.1B*		C03:13880644:13883273:+	2630	1098	365	5.61	−0.549	12–11	Nuclear
12	BnaA05g03780D	*BnASHH3.2A*		A05:2018708:2020905:+	2198	1098	365	6.37	−0.519	11–10	Nuclear
13	BnaC04g03390D	*BnASHH3.2B*		C04:2408832:2411322:+	2491	1134	377	6.87	−0.516	12–11	Nuclear
14	BnaA07g18580D	*BnASHH4.1A*		A07:15109224:15112095:+	2872	966	321	9.32	−0.679	9–8	Nuclear
15	BnaC06g17610D	*BnASHH4.1B*		C06:20191156:20193591:+	2436	966	321	9.2	−0.666	9–8	Nuclear
16	BnaA09g38210D	*BnASHH4.2A*		A09:27345059:27347078:+	2020	1044	347	8.94	−0.493	11–10	Nuclear
17	BnaC08g30180D	*BnASHH4.2B*		C08:30147366:30149394:+	2029	1083	360	9.1	−0.521	12–11	Nuclear
18	BnaA08g12860D	*BnASHR3.1A*		A08:11310674:11313478:−	2805	1485	494	8.24	−0.487	11–10	Extracellular
19	BnaC03g67870D	*BnASHR3.1B*		C03:57480155:57482886:−	2732	1488	495	8.59	−0.464	11–10	Extracellular
20	BnaA07g33020D	*BnASHH1.1A*		A07:22719804:22721972:−	2169	1386	461	5.07	−0.682	8–7	Nuclear
21	BnaC06g37540D	*BnASHH1.1B*		C06:35510189:35512505:−	2317	1386	461	5.03	−0.656	8–7	Nuclear
22	BnaA07g17090D	*BnASHH1.2A*		A07:14363518:14367606:−	4089	774	257	5.1	−0.434	5–4	Extracellular
23	BnaA07g33460D	*BnASHH2.1A*		A07:22947562:22954942:−	7381	4974	1657	5.69	−0.765	17–16	Nuclear
24	BnaC06g38010D	*BnASHH2.1B*		C06:35772518:35779988:−	7471	4974	1657	5.94	−0.761	18–17	Nuclear
25	BnaA04g18180D	*BnATX1.1A*	III	A04:14637597:14643717:+	6121	3093	1030	8.54	−0.477	24–23	Nuclear
26	BnaC04g42250D	*BnATX1.1B*		C04:42853666:42859699:+	6034	3105	1034	8.31	−0.48	24–23	Nuclear
27	BnaA09g50210D	*BnATX2.1A*		A09:33252913:33259540:−	6628	3189	1062	8.38	−0.514	26–25	Nuclear
28	BnaC08g44440D	*BnATX2.1B*		C08:37672655:37679549:+	6895	3342	1113	8.59	−0.504	25–24	Nuclear
29	BnaA07g19000D	*BnATX3.1A*		A07:15365794:15371466:-	5673	2895	964	7.91	−0.534	26–25	Nuclear
30	BnaC06g18140D	*BnATX3.1B*		C06:20544144:20550243:-	6100	2934	977	8.14	−0.577	25–24	Nuclear
31	BnaA03g48900D	*BnATX4.1A*		A03:25126743:25131753:+	5011	2874	957	8.98	−0.493	22–21	Nuclear
32	BnaC07g41090D	*BnATX4.1B*		C07:41096336:41101563:+	5228	2751	916	9	−0.524	26–25	Nuclear
33	BnaA10g06740D	*BnATX5.1A*		A10:5263397:5269324:+	5928	2925	974	8.76	−0.595	23–22	Nuclear
34	BnaC09g29770D	*BnATX5.1B*		C09:32512813:32518626:+	5814	2925	974	8.78	−0.61	23–22	Nuclear
35	BnaA01g18730D	*BnATXR3.1A*		A01:10213817:10224101:−	10,285	6897	2298	7.63	−0.773	21–20	Nuclear
36	BnaC01g42140D	*BnATXR3.1B*		C01_random:1186565:1187739:-	1175	882	293	4.76	−0.626	4–3	Cytoplasm
37	BnaA05g13380D	*BnATXR3.2A*		A05:8123162:8131857:+	8696	5154	1717	6.05	−0.576	20–19	Nuclear
38	BnaC06g11340D	*BnATXR3.2B*		C06:13248380:13258468:−	10,089	6261	2086	6.29	−0.796	21–20	Nuclear
39	BnaA09g16090D	*BnATXR7.1A*		A09:9526683:9533922:−	7240	3867	1288	8.5	−0.562	18–17	Nuclear
40	BnaC09g16850D	*BnATXR7.1B*		C09:13648653:13652429:−	3777	1470	489	9.55	−0.665	10–9	Nuclear
41	BnaA02g00160D	*BnATXR5.1A*	IV	A02:59526:61901:-	2376	1170	389	8.45	−0.46	5–4	Nuclear
42	BnaC02g00720D	*BnATXR5.1B*		C02:298303:300331:+	2029	1068	355	7.04	−0.316	6–5	Nuclear
43	BnaA10g22360D	*BnATXR5.2A*		A10:15105735:15107523:+	1789	1164	387	8.84	−0.636	6–5	Nuclear
44	BnaC09g46870D	*BnATXR5.2B*		C09:46520440:46522899:+	2460	1158	385	8.91	−0.669	6–5	Nuclear
45	BnaA02g37130D	*BnATXR6.1A*		A02_random:1596786:1601614:+	4829	1653	550	8.92	−0.391	10–9	Nuclear
46	BnaCnng48300D	*BnATXR6.1B*		Cnn_radom:47593540:47595192:+	1653	1029	342	9	−0.492	6–5	Nuclear
47	BnaA06g26870D	*BnATXR6.2A*		A06:18437953:18439411:−	1459	1038	345	8.83	−0.446	6–5	Nuclear
48	BnaC07g30100D	*BnATXR6.2B*		C07:34718609:34720078:+	1470	1038	345	8.75	−0.487	6–5	Nuclear
49	BnaA03g01550D	*BnSUVH1.1A*	V	A03:700506:703243:−	2738	1938	645	8.48	−0.432	1–0	Nuclear
50	BnaC03g01840D	*BnSUVH1.1B*		C03:886615:888912:−	2298	1935	644	8.63	−0.44	1–0	Nuclear
51	BnaA10g25310D	*BnSUVH1.2A*		A10:16309620:16314421:+	4802	3039	1012	7.06	−0.398	5–4	Nuclear
52	BnaC09g50310D	*BnSUVH1.2B*		C09:48207580:48210227:+	2648	1842	613	6.31	−0.566	4–3	Nuclear
53	BnaC02g02520D	*BnSUVH1.3B*		C02:1111629:1114084:+	2456	2169	722	8.68	−0.43	3–2	Chloroplast
54	BnaC09g50300D	*BnSUVH1.4B*		C09:48204638:48207123:+	2486	1968	655	8.64	−0.458	3–2	Nuclear
55	BnaA07g30600D	*BnSUVH3.1A*		A07:21627723:21630514:+	2792	1974	657	8.31	−0.415	2–1	Nuclear
56	BnaC06g43880D	*BnSUVH3.1B*		C06_random:3270281:3273083:+	2803	1980	659	8.59	−0.421	2–1	Nuclear
57	BnaA07g22850D	*BnSUVH3.2A*		A07:17311770:17314665:−	2896	2001	666	8.31	−0.454	1–0	Nuclear
58	BnaC06g23810D	*BnSUVH3.2B*		C06:25579994:25583205:−	3212	2010	669	8.31	−0.465	1–0	Nuclear
59	BnaC06g43300D	*BnSUVH3.3B*		C06_random:2755627:2758345:−	2719	2010	669	8.31	−0.456	1–0	Nuclear
60	BnaAnng14120D	*BnSUVH7.1A*		Ann_random:15255512:15258054:−	2543	2382	793	5.44	−0.558	2–1	Nuclear
61	BnaC06g28920D	*BnSUVH7.1B*		C06:30089672:30096121:+	6450	5571	1466	4.6	−0.469	3–2	Nuclear
62	BnaA06g11960D	*BnSUVH7.2A*		A06:6207143:6216147:+	9005	2688	895	5.37	−0.582	5–4	Nuclear
63	BnaA06g11990D	*BnSUVH7.3A*		A06:6229288:6231537:+	2250	2097	698	5.41	−0.577	3–2	Nuclear
64	BnaAnng21540D	*BnSUVH7.4A*		Ann_radom:24010960:24013125:+	2166	2145	714	5.77	−0.564	2–1	Nuclear
65	BnaA03g04610D	*BnSUVH4.1A*		A03:2148052:2152060:+	4009	1815	604	8.1	−0.594	14–13	Nuclear
66	BnaC03g06140D	*BnSUVH4.1B*		C03:2974553:2979472:+	4920	1812	603	7.71	−0.615	14–13	Nuclear
67	BnaA05g10320D	*BnSUVH2.1A*		A05:5614940:5616640:−	1701	1701	566	8.71	−0.227	1–0	Mitochondria
68	BnaC04g11240D	*BnSUVH2.1B*		C04:8711809:8713467:−	1659	1659	552	6.48	−0.278	1–0	Nuclear
69	BnaA04g19330D	*BnSUVH2.2A*		A04:15264117:15266030:+	1914	1914	637	7.15	−0.347	1–0	Nuclear
70	BnaC04g43670D	*BnSUVH2.2B*		C04:43996787:43998700:+	1914	1914	637	7.84	−0.351	1–0	Nuclear
71	BnaA09g34050D	*BnSUVH9.1A*		A09:25038028:25040666:-	2639	1896	631	5.46	−0.382	2–1	Nuclear
72	BnaC08g24970D	*BnSUVH9.1B*		C08:26899934:26902602:−	2669	1902	633	5.44	−0.382	2–1	Nuclear
73	BnaA05g32760D	*BnSUVR3.1A*		A05:22388231:22390304:-	2074	1011	336	8.18	−0.185	2–1	Extracellular
74	BnaC05g48060D	*BnSUVR3.1B*		C05:42732413:42734037:−	1625	1011	336	8.32	−0.144	2–1	Nuclear
75	BnaA05g08770D	*BnSUVH5.1A*		A05:4846708:4848861:−	2154	2154	717	8.72	−0.593	1–0	Nuclear
76	BnaC04g10170D	*BnSUVH5.1B*		C04:7828228:7830906:−	2679	2166	721	8.88	−0.609	1–0	Nuclear
77	BnaA04g13190D	*BnSUVH6.1A*		A04:11068672:11071386:−	2715	2241	746	8.64	−0.653	3–2	Nuclear
78	BnaC04g35290D	*BnSUVH6.1B*		C04:36693689:36695998:−	2310	2277	758	8.7	−0.656	2–1	Nuclear
79	BnaA02g22450D	*BnSUVR2.1A*		A02:14963608:14967065:+	3458	1923	640	5.18	−0.492	11–10	Nuclear
80	BnaC02g30730D	*BnSUVR2.1B*		C02:32562070:32566295:+	4226	2355	784	5.68	−0.449	12–11	Nuclear
81	BnaA06g36410D	*BnSUVR2.2A*		A06:23853703:23857119:−	3417	2118	705	5.27	−0.465	11–10	Nuclear
82	BnaC07g17530D	*BnSUVR2.2B*		C07:23844342:23848092:+	3751	2058	685	5.32	−0.501	11–10	Nuclear
83	BnaA03g28550D	*BnSUVR4.1A*		A03:13930218:13933493:+	3276	1404	467	7.94	−0.622	8–7	Nuclear
84	BnaC03g33690D	*BnSUVR4.1B*		C03:20516321:20518949:+	2629	1419	472	7.1	−0.632	8–7	Nuclear
85	BnaA05g33030D	*BnSUVR4.2A*		A05:22506210:22509857:−	3648	1674	557	5.82	−0.582	9–8	Nuclear
86	BnaC05g47290D	*BnSUVR4.2B*		C05:42343619:42349146:−	5528	2487	828	5.82	−0.382	12–11	Extracellular
87	BnaA04g13850D	*BnSUVR5.1A*		A04:11704476:11710607:+	6132	4101	1366	6.25	−0.473	11–10	Nuclear
88	BnaA09g41880D	*BnSUVR5.2A*		A09:29184943:29188659:−	3717	1479	492	5.6	−0.481	11–10	Nuclear
89	BnaC04g56280D	*BnSUVR5.1B*		C04_random:3971855:3978126:+	6272	4101	1366	6.25	−0.486	11–10	Nuclear
90	BnaA07g02410D	*BnASHR1.1A*	VI	A07:2033534:2037030:+	3497	1452	483	7.73	−0.269	14–13	Nuclear
91	BnaCnng42550D	*BnASHR1.1B*		Cnn_random:41655054:41658605:−	3552	1452	483	7.97	−0.259	14–13	Nuclear
92	BnaA09g09920D	*BnASHR2.1A*		A09:5047113:5048261:+	1149	1149	382	4.49	−0.368	1–0	Nuclear
93	BnaA09g29220D	*BnATXR1.1A*		A09:21881217:21882842:-	1626	1626	541	6.16	−0.34	1–0	Cytoplasmic
94	BnaCnng33180D	*BnATXR1.1B*		Cnn_radom:31543831:31545456:+	1626	1626	541	6.97	−0.354	1–0	Cytoplasmic
95	BnaA05g19420D	*BnATXR2.1A*		A05:14796293:14799111:+	2819	1311	436	4.8	−0.258	14–13	Nuclear
96	BnaCnng26330D	*BnATXR2.1B*		Cnn_random:24846860:24849927:−	3068	1416	471	8.84	−0.195	15–14	Nuclear
97	BnaAnng0177D	*BnATXR4.1A*		Ann_random:988229:990161:+	1933	990	329	7.44	−0.094	7–6	Extracellular
98	BnaC02g01930D	*BnATXR4.1B*		C02:841583:843636:-	2054	990	329	6.96	−0.082	7–6	Extracellular
99	BnaA05g17880D	*BnaA05g17880D*		A05:12965688:12970193:+	4506	2361	786	6.59	−0.141	14–13	Nuclear
100	BnaCnng44980D	*BnaCnng44980D*		A09_random:246310:248642:−	5360	2748	915	6.68	−0.101	16–15	Nuclear
101	BnaA09g52100D	*BnaA09g52100D*		Cnn_random:1772446:1777817:−	2333	1677	558	5.83	−0.027	4–3	PM
102	BnaCnng01720D	*BnaCnng01720D*		Cnn_radom:44087053:44092412:+	5372	2298	765	7.81	−0.37	7–6	Nuclear/PM
103	BnaCnng08960D	*BnSETD1.1B*	VII	Cnn_random:8235749:8239114:+	3366	1716	571	4.78	−0.203	15–14	Cytoplasmic
104	BnaA09g45900D	*BnSETD2.1A*		A09:31291564:31293629:+	2066	1455	484	4.82	−0.171	6–5	Chloroplast
105	BnaC08g39970D	*BnSETD2.1B*		C08:35400386:35402532:+	2147	1458	485	4.78	−0.161	6–5	chloroplast
106	BnaA09g28320D	*BnSETD3.1A*		A09:21211771:21213718:−	1948	1428	475	5.81	−0.101	5–4	Mitochondria/Cytoplasmic
107	BnaAnng22450D	*BnSETD3.2A*		Ann_radom:25207576:25208943:+	1368	1101	366	5.06	−0.119	4–3	Cytoplasmic
108	BnaC05g20890D	*BnSETD3.1B*		C05:14486930:14488943:+	2014	1416	471	4.87	−0.294	5–4	Mitochondria
109	BnaA07g01600D	*BnSETD4.1A*		A07:1248812:1251691:+	2880	1617	538	4.87	−0.294	10–9	Cytoplasmic
110	BnaC07g02950D	*BnSETD4.1B*		C07:3766179:3768855:−	2677	1626	541	4.76	−0.312	10–9	Cytoplasmic
111	BnaC01g39740D	*BnSETD5.1B*		C01:38283208:38286353:-	3146	1524	507	8.66	−0.388	12–11	Chloroplast
112	BnaA09g34820D	*BnSETD6.1A*		A09:25444044:25449106:−	5063	1404	467	6.84	−0.203	15–14	Mitochondria/PM
113	BnaC08g25980D	*BnSETD6.1B*		C08:27486308:27488874:−	2567	930	309	5.12	−0.121	11–10	PM/Mitochondria/ Chloroplast
114	BnaA09g35960D	*BnSETD7.1A*		A09:26136452:26138636:+	2185	1242	413	4.5	−0.326	5–4	Nuclear/Cytoplasmic/ER
115	BnaA09g35970D	*BnSETD7.2A*		A09:26139780:26142012:+	2233	1548	515	4.9	−0.311	5–4	Nuclear/Cytoplasmic
116	BnaC08g27460D	*BnSETD7.1B*		C08:28436643:28438862:+	2220	1461	486	4.72	−0.291	5–4	Nuclear/Cytoplasmic
117	BnaA03g14450D	*BnSETD8.1A*		A03:6658037:6659546:+	1510	798	265	6.67	−0.215	9–8	Chloroplast
118	BnaA04g22390D	*BnSETD8.2A*		A04:16863871:16865991:+	2121	999	332	5.55	−0.297	12–11	Nuclear/Cytoplasmic
119	BnaA08g30390D	*BnSETD8.3A*		A08_random:1468364:1476519:+	8156	2034	677	5.74	−0.378	17–16	Nuclear
120	BnaC03g63720D	*BnSETD8.1B*		C03:53197435:53200160:+	2726	1455	484	5.59	−0.278	12–11	Cytoplasmic/Mitochondrial/Chloroplast
121	BnaA02g03480D	*BnSETD10.1A*		A02:1543050:1544888:+	1839	1446	481	5.16	−0.338	6–5	Cytoplasmic
122	BnaC02g07170D	*BnSETD10.1B*		C02:3795126:3796969:+	1844	1440	479	5.32	−0.375	6–5	Cytoplasmic

## Data Availability

The corresponding data have been shown in Supplementary Materials.

## Supplementary Materials

The following are available online at https://www.mdpi.com/article/10.3390/ijms23041936/s1.
