# Supplementary material for: Genome-Wide Identification and Characterization of SET Domain Family Genes in Brassica napus L
Source: Int J Mol Sci. 2022 Feb 9;23(4):1936. doi: 10.3390/ijms23041936 (PMC8879272; doi:10.3390/ijms23041936)
Supplement: Supplementary file 1 [file ijms-23-01936-s001.zip › supplimentary figures.pdf]

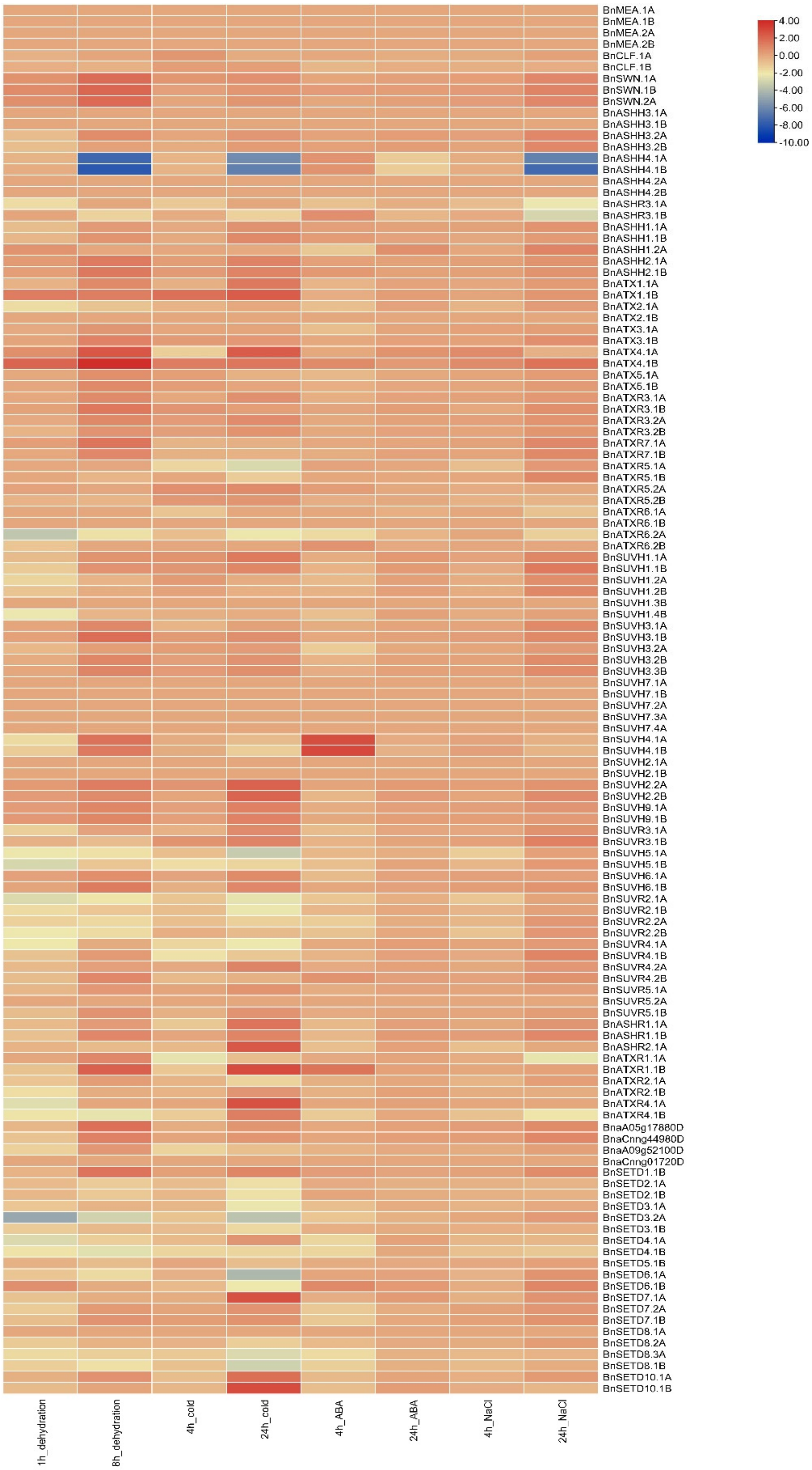

Figure S1. Heat map of in silico expression (Log2 fold change) of *BnSDG*s under dehydration, cold, ABA and salinity stress. The h reperesents the sample collection at specific hours after the treatments.

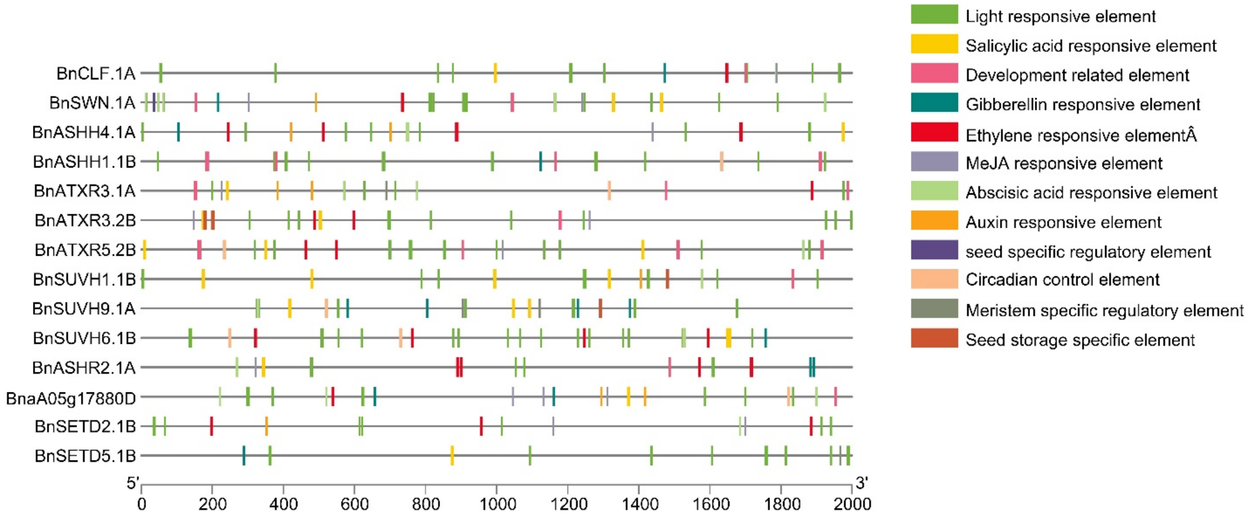

Figure S2. The potential cis-regulatory elements in the 2kb upstream coding region of 15 candidate *BnSDGs*.
